# Quality indices for topic model selection and evaluation: a literature review and case study

**DOI:** 10.1186/s12911-023-02216-1

**Published:** 2023-07-22

**Authors:** Christopher Meaney, Therese A. Stukel, Peter C. Austin, Rahim Moineddin, Michelle Greiver, Michael Escobar

**Affiliations:** 1 Department of Family and Community Medicine, University of Toronto, 500 University Ave, Toronto, ON M5G1V7 Canada; 2 Institute of Health Policy, Management and Evaluation, ICES, University of Toronto, Toronto, Canada; 3 Dalla Lana School of Public Health, University of Toronto, Toronto, Canada

**Keywords:** Non-negative matrix factorization, Topic model, Internal validation, Cross-validation, Stability analysis, Clinical text data, Electronic medical record

## Abstract

**Background:**

Topic models are a class of unsupervised machine learning models, which facilitate summarization, browsing and retrieval from large unstructured document collections. This study reviews several methods for assessing the quality of unsupervised topic models estimated using non-negative matrix factorization. Techniques for topic model validation have been developed across disparate fields. We synthesize this literature, discuss the advantages and disadvantages of different techniques for topic model validation, and illustrate their usefulness for guiding model selection on a large clinical text corpus.

**Design, setting and data:**

Using a retrospective cohort design, we curated a text corpus containing 382,666 clinical notes collected between 01/01/2017 through 12/31/2020 from primary care electronic medical records in Toronto Canada.

**Methods:**

Several topic model quality metrics have been proposed to assess different aspects of model fit. We explored the following metrics: reconstruction error, topic coherence, rank biased overlap, Kendall’s weighted tau, partition coefficient, partition entropy and the Xie-Beni statistic. Depending on context, cross-validation and/or bootstrap stability analysis were used to estimate these metrics on our corpus.

**Results:**

Cross-validated reconstruction error favored large topic models (K ≥ 100 topics) on our corpus. Stability analysis using topic coherence and the Xie-Beni statistic also favored large models (K = 100 topics). Rank biased overlap and Kendall’s weighted tau favored small models (K = 5 topics). Few model evaluation metrics suggested mid-sized topic models (25 ≤ K ≤ 75) as being optimal. However, human judgement suggested that mid-sized topic models produced expressive low-dimensional summarizations of the corpus.

**Conclusions:**

Topic model quality indices are transparent quantitative tools for guiding model selection and evaluation. Our empirical illustration demonstrated that different topic model quality indices favor models of different complexity; and may not select models aligning with human judgment. This suggests that different metrics capture different aspects of model goodness of fit. A combination of topic model quality indices, coupled with human validation, may be useful in appraising unsupervised topic models.

**Supplementary Information:**

The online version contains supplementary material available at 10.1186/s12911-023-02216-1.

## Background

An increasing share of modern human communication is captured in digital text format [1]. The increasing digitization of communication creates a demand for computational and statistical methods to facilitate exploration, understanding and extraction of meaningful insights from these voluminous and complex text data sources.

Topic models represent a class of statistical techniques for summarizing large text corpora. These models represent a document as arising from an admixture of latent topical vectors. The k = 1…K latent topical vectors describe the thematic content of the corpus using a set of semantically correlated word clusters. An admixing parameter (of dimension K) expresses the extent to which a particular document displays an affinity for a particular topic. Numerous statistical algorithms exist for estimating a topic model. One of the earliest techniques involved representing a text corpus as a document term matrix (DTM) and decomposing the resulting large sparse DTM via singular value decomposition – a methodology coined Latent Semantic Analysis (LSA) [2–4]. To facilitate improved interpretation, non-negative matrix factorization (NMF) has been employed to decompose the DTM. NMF summarizes an input DTM in terms of a low-rank outer product decomposition; however, in NMF the statistical decomposition forces non-negativity constraints on row and column bases, yielding an additive parts-based interpretation [5–7]. To enhance interpretation of how complex document collections emerge, probabilistic extensions of LSA/NMF were developed, including probabilistic latent semantic indexing (pLSI) [8] and latent Dirichlet allocation (LDA) [9–12].

As with other unsupervised machine learning algorithms, post-hoc evaluation, validation, and criticism of fitted topic models is encouraged, albeit challenging. Depending on the variant of topic model fit to a given document collection, several hyper-parameters need to be tuned to achieve a meaningful summarization of the corpus. A common hyper-parameter employed in all the aforementioned topic models is the number of topics (k = 1,2,3,…K), a discrete positive hyper-parameter which governs model complexity. Specification of too few topics often yields a noisy thematic characterization, where learned topics are broad in scope (i.e. words loading highly on a given topic are not semantically correlated); whereas, specification of too many topics often results in over-clustering (a phenomena whereby semantically related topics are redundantly repeated in the summarization). Topic model validity indices are post-hoc quantitative metrics which can be used to guide the analyst towards aspects of a “good fitting” model. A wide variety of internal model quality indices have been proposed across disparate statistical research fields.

The primary objective of this manuscript is to review and synthesize literature surrounding modern topic model validity indices. As a secondary objective, we fit several topic models and apply different topic model quality indices to a large corpus of clinical notes collected from primary care electronic medical records from Toronto, Canada. We comment on the ability of these quality indices to guide analysts towards a consensus model. We critically appraise the different topic model quality indices and investigate how different metrics assess different aspects of model stability, robustness, and goodness of fit.

### Literature review

#### Non-negative matrix factorization topic models

In this study, we fit a non-negative matrix factorization (NMF) topic model to an input document term matrix (DTM). The DTM is a large sparse matrix with d = 1…D rows (a single row for each document/note in the corpus) and v = 1…V columns (a single column for each word/token in the empirical vocabulary). Each element of the DTM $({X}_{dv})$ is a count random variable, denoting the number of times word/token (v) occurs in document (d). NMF factorizes the D*V dimensional DTM into two latent sub-matrices of dimension D*K ($\theta$) and K*V ($\phi$). The DTM (X) consists of non-negative integers (i.e. word frequency counts); whereas, the learned matrices ($\theta$,$\phi$) consist of non-negative real values.

$$\left(\begin{array}{cc}\begin{array}{cc}{X}_{11}& \cdots \\ \vdots & \end{array}& \begin{array}{cc}\cdots & {X}_{1V}\\ & \vdots \end{array}\\ \begin{array}{cc}\vdots & \\ \vdots & \\ {X}_{D1}& \cdots \end{array}& \begin{array}{cc}& \vdots \\ & \vdots \\ \cdots & {X}_{DV}\end{array}\end{array}\right) \approx \left(\begin{array}{cc}\begin{array}{c}{\theta }_{11}\\ \vdots \end{array}& \begin{array}{cc}\cdots & {\theta }_{1K}\\ & \vdots \end{array}\\ \begin{array}{c}\vdots \\ \vdots \\ {\theta }_{D1}\end{array}& \begin{array}{cc}& \vdots \\ & \vdots \\ \cdots & {\theta }_{DK}\end{array}\end{array}\right)\left(\begin{array}{cc}\begin{array}{cc}{\phi }_{11}& \cdots \end{array}& \begin{array}{cc}\cdots & {\phi }_{1V}\end{array}\\ \begin{array}{cc}\vdots & \\ {\phi }_{K1}& \cdots \end{array}& \begin{array}{cc}& \vdots \\ \cdots & {\phi }_{KV}\end{array}\end{array}\right)$$

Many suitable objective functions have been proposed for learning the NMF latent matrices (θ,$\phi$). We introduce a general NMF objective function below.

$$L\equiv \underset{\left\{\theta \ge 0, \phi \ge 0\right\}}{\mathrm{argmin}}\sum_{d=1}^{D}\sum_{v=1}^{V}{w}_{\left\{d,v\right\}}*f\left({X}_{\left\{d,v\right\}}; \sum_{k=1}^{K}{\theta }_{\{d,k\}}{\phi }_{\{k,v\}}\right)+\Lambda \left({\theta }_{d,k}, {\phi }_{k,v}\right)$$

The objective function specifies that the observed elements of the DTM are approximated by a K-dimensional bilinear form ($\sum_{k=1}^{K}{\theta }_{\left\{d,k\right\}}{\phi }_{\left\{k,v\right\}}$). The user must specify the dimension of the latent space (K). The model can be adapted to different data generating mechanisms via the choice of loss function ($f\left({X}_{\left\{d,v\right\}}; \sum_{k=1}^{K}{\theta }_{\left\{d,k\right\}}{\phi }_{\left\{k,v\right\}}\right)$). Arbitrary weighting of data points can be accommodated through ${w}_{\left\{d,v\right\}}$ (examples of weighted NMF models are given in Udell et al. [13]). Regularization ($\Lambda \left({\theta }_{d,k}, {\phi }_{k,v}\right)$) can be introduced to achieve parameter estimates with desirable properties (e.g. sparsity, smoothness, minimum volume, etc.). Seminal articles on NMF include Paatero & Tapper [14] and Lee & Seung [5, 6]. Surveys of NMF and low rank models are given in Berry [15] and Udell et al. [13].

When using NMF for topic modelling, a sparse D*V dimensional DTM (X) is factored into two non-negative real matrices: a D*K dimensional matrix of per-document topic parameters ($\theta$) and a K*V dimensional matrix of per-topic word parameters ($\phi$). The NMF model imposes non-negativity constraints on the estimates of the latent matrices ($\theta$ and $\phi$). Post-hoc, one can normalize the row-vectors constituting both $\theta$ and $\phi$, dividing by their respective row-sums. The resulting normalized vectors can be interpreted as compositional/probability vectors (i.e. each normalized row of $\theta$ and $\phi$ contains non-negative entries which sum to one). Row vectors of the matrix $\phi$ encode a set of k = 1…K per-topic word probabilities (estimated over a discrete set of v = 1…V words in our corpus). Row vectors of the matrix $\theta$ encode a set of d = 1…D per-document topic proportions (estimated over a discrete set of k = 1…K latent dimensions), encoding the affinity a given document has for a particular topic.

### Quality indices/metrics for evaluating NMF topic models

A topic model validity index is a numeric metric/score used to guide selection of an “optimal” topic model fitted to a given document collection. The choice of an “optimal” model is context dependent and, in many cases, may even represent a nebulous concept [16, 17]. As a result of the difficulty associated with a priori defining the attributes of an optimal performing topic model, different validity indices have been developed across different statistical research communities, which highlight different aspects of model goodness of fit. Four broad classes of topic model quality indices will be introduced: 1) metrics which emphasize model fit (i.e. residual error or reconstruction error), 2) metrics which focus on evaluation of the per-topic distribution over words matrix $({\phi }_{\{k,v\}})$, 3) metrics which prioritize evaluation of the per-document distribution over topics matrix (${\theta }_{\{d,k\}}$), and 4) metrics which simultaneously combine evaluation of ${\theta }_{\{d,k\}}$ and ${\phi }_{\{k,v\}}.$

Each of the topic quality indices discussed are examples of internal validation indices [18, 19]. Internal indices construct a validation score using only data available during the topic model fitting process. These internal indices can be contrasted with external validation indices. An external validation index uses information collected via the same sampling process that generated the original DTM; however, it is external to the topic model fitting/estimation algorithm. For example, it is common to evaluate a topic model in terms of the ability of the latent topical basis (particularly the D*K matrix of per-document topic weights, θ) to predict an external target vector in a regression/classification context.

A final approach to validating topic models involves subjective human interpretation/validation.

Matthews [20] describe “eyeballing” as a common approach to validating (or ascribing meaning) to fitted topic models. Using the “eyeballing” method, researchers fit several topic models to an observed document collection (over a pre-determined hyper-parameter grid) and subjectively label learned topic distributions by inspection of high-loading words/tokens (from $\phi$) or high-loading documents (from $\theta$). This subjective human-centric approach to topic model validation parallels that of face validity checks or social validity checks used in qualitative content analyses [21]. Doogan et al. [17] are also proponents of a more exhaustive approach to human-in-the-loop topic model validation where both the latent topic vectors and the per-document topic weights are simultaneously evaluated.

### Monte Carlo cross validation on reconstruction error metrics

Cross validation is a commonly employed methodology for estimating model performance and conducting model validation/selection [22]. Several challenges arise when cross validating a matrix factorization topic model. The input data structure in NMF topic modelling is a sparse high-dimensional DTM. When performing cross-validation in the context of NMF topic modelling we do not want to hold-out an entire row/column of the DTM. If an entire row/column index of the DTM is held-out for validation/testing (in a k-fold cross-validation scheme), then the training algorithm will never learn an embedding/basis over the held-out row/column indices. As such, simple k-fold cross-validation schemes are not amenable for cross-validating an NMF topic model.

Wold [23] discusses several hold-out schemes relevant to cross-validating matrix factorization models. One scheme holds-out individual elements/indices (d,v) at random from the DTM, in such a manner that no entire row/column is left out of the training process. Wold [23] introduces an alternative cross-validation scheme which holds out diagonal bands of the DTM, again ensuring that no entire row/column is excluding from the training process. Owens et al. [24] extends the idea, holding out contiguous blocks of rows/columns, again ensuring no entire/row column is held out of the DTM during the training and cross-validation process. Bro et al. [25] review cross-validation in the context of matrix factorization problems. Lastly, if the matrix (or DTM) being sampled is dense many of the above cross validation schemes are trivial to implement; however, if the matrix is sparse (as is the case with many DTMs), checks on the validity of the hold-out process need to be carefully implemented.

In this study we employ a Monte Carlo cross-validation scheme similar to Wold [23]. We represent the DTM using a sparse triplet/coordinate-format data structure. We randomly sample 80% of data elements for inclusion in the training process, and 20% of data elements are held-out for inclusion in the validation process. Sampling is conducted without replacement. When sampling (d,v,x) triples for inclusion in the training sample, we write assertions to check that all row indices (d = 1…D) and all column indices (v = 1…V) are included in the training sample. If a randomly generated training sample excludes an entire row/column index) then the Monte Carlo cross-validation sample is rejected as invalid. We repeat the random sampling process five times (noting that sampling (d,v,x) triples from a large/sparse DTM is computationally expensive). We fit five independent NMF topic models to each Monte Carlo cross-validation training sample, estimate reconstruction error on each of the training/validation samples, and average the reconstruction errors over held-out validation samples.

### Bootstrap stability analysis using topic coherence metrics

Stability analysis is a generic methodology for evaluating the quality of a fitted unsupervised machine learning model. The methodology proceeds by drawing bootstrap samples (i.e. sampling with replacement) from the original data structure; a model is fitted to each bootstrap replicate dataset, and its quality/stability is evaluated [26, 27]. The specifics of the methodology are dependent on the topic model quality metric used in the analysis. In this section we discuss stability analysis in the context of topic coherence metrics.

Topic coherence metrics represent a family of scoring functions used to quantify the semantic correlation of word/token lists. For a given topic model, consider the top-P words/tokens loading most highly on a specific topical vector (k). Typically P is chosen to be a small integer value (P = {5, 10, 25, etc.}). Topical coherence is estimated by constructing $$\left(\begin{array}{c}P\\ 2\end{array}\right)$$ co-occurrence scores between each pair of words/tokens in the top-P list. The co-occurrence scores are based on word frequency co-occurrence counts in the observed document collection (although external corpora may also be introduced for scoring). For each topic k = 1…K, an estimate of topical coherence is obtained. Averaging over k = 1…K topics in a fitted topic model results in an overall coherence score for the model fit. These scores can then be compared across b = 1…B bootstrap replicate samples to assess model stability. In this manuscript we consider two metrics: the UCI scoring metric [28, 29] and the UMASS scoring metric [30]. Development of topic coherence scores remains a popular area of research and a review on available topic coherence metrics is discussed in Roder et al. [31].

The UCI and UMASS scores are given below. The quantities describe the marginal or joint occurrence probabilities of words/tokens in the empirical corpus (although any arbitrarily chosen external corpus could be used to estimate the marginal/joint probabilities of a word occurrence).

$${score}_{UCI}\left({w}_{i},{w}_{j}\right)=\mathrm{log}\left(\frac{P\left({w}_{i},{w}_{j}\right)}{P\left({w}_{i}\right)P\left({w}_{j}\right)}\right)$$

$${score}_{UMASS}\left({w}_{i},{w}_{j}\right)=\mathrm{log}\left(\frac{P\left({w}_{i},{w}_{j}\right)+1}{P\left({w}_{i}\right)}\right)$$

### Stability analysis using set based agreement metrics

Set based agreement metrics can also be employed to assess topic model stability. Under this approach to stability analysis, we begin by drawing b = 1…B bootstrap replicate samples from the original DTM (i.e. sampling with replacement). The goal is to compare agreement between top-P lists of semantically aligned topics fitted under different models. If there exist B bootstrap samples drawn for the stability analysis, then there exist $$\left(\begin{array}{c}B\\ 2\end{array}\right)$$ models to compare, each of complexity K. For any given per-topic word distribution (indexed k = 1…K), and any two models (M_s_ and M_t_ from bootstrap samples b_s_ and b_t_ respectively – for {s,t} = 1…B) the goal is to pair-wise compare the two resulting top-P lists (over k = 1…K latent dimensions in the model). There exist numerous metrics for comparing top-P lists of discrete items, and these types of metrics are reviewed in Fagin et al. [32]. In this study, we will use a particular set-based agreement metric, rank biased overlap (RBO) [33].

An inherent challenge associated with this variant of stability analysis involves the exchangeability of the learned topics from a fitted NMF model. That is, the ordering of the topical basis in the low-rank reconstruction is arbitrary. As such, even if two NMF topic models of the same latent dimension are fitted to a dataset (or two different bootstrap datasets) there is no guarantee that semantically related topics occur in the same (arbitrary) ordering across different model fits. However, it is possible to align the learned topical bases (${\phi }_{s}$) and (${\phi }_{t}$) across the two bootstrap datasets. Alignment of the respective topical matrices is a type of linear-sum-assignment problem. In other words, the goal is to learn an optimal K*K permutation matrix (π ε П) such that the Frobenius norm error between the topical matrices (${\phi }_{s}$) and (${\phi }_{t}$) is minimized. Solving such a problem can be approached using the Hungarian algorithm [34, 35; see also Appendix A].

$$\underset{\pi \epsilon\Pi }{\mathrm{argmin}}{\Vert {\phi }_{s}- \pi {\phi }_{t}\Vert }_{2}$$

Given two aligned topical matrices, from two NMF model fits, on two distinct bootstrap datasets, we proceed to estimating the RBO agreement metric between the two top-P token/word lists (S and T).

Mathematically RBO is defined below. The metric measures the weighted average agreement between two top-P sets. The metric lives on the space [0,1] with zero indicating no agreement between top-P lists, and one indicating complete agreement. The RBO score is a function of a tunable hyper-parameter (z $\in$ (0,1)) which determines how the score prioritizes agreement over top-P list depth. Smaller values of z favor top-weighted elements in the list, and when z = 0 (in the limit) only the first item in the pair of lists are compared. A_P_ measures the fractional/proportional agreement of the lists (S and T) up to depth-P. An example of top-5 agreement between word lists is given below (Table 1).

**Table 1:** Example of average set-based agreement calculated over different top-P depths (*P* = 1,2,3,4,5)

Depth (P)	Tokens in Bootstrap Sample-S at Depth-P	Tokens in Bootstrap Sample-T at Depth-P	Intersection of Tokens in Samples S and T	Fraction Agreement (A_P_)
1	Cough	Flu	{}	0/2 (0)
2	Cough, Cold	Flu, Cold	{Cold}	1/3 (33.3)
3	Cough, Cold, Flu	Flu, Cold, Cough	{Cough, Cold, Flu}	3/3 (100.0)
4	Cough, Cold, Flu, Fever	Flu, Cold, Cough, Phlegm	{Cough, Cold, Flu}	3/5 (60.0)
5	Cough, Cold, Flu, Fever, Phlegm	Flu, Cold, Cough, Phlegm, Fever	{Cough, Cold, Flu, Fever, Phlegm}	5/5 (100.0)

$$RBO\left(S,T,P,A\right)=(1-z)\sum_{P=1}^{\infty }{z}^{P-1}{A}_{P}$$

We note that using an identical strategy as defined above, the RBO metric could also be used to assess the stability of returned top-P document-lists from the matrix of per-document topic proportions (θ).

### Stability analysis using rank based correlation metrics

Another commonly encountered metric for comparing top-P lists is Kendall’s Tau statistic, a measure of rank-based correlation [36] ranging between [-1, + 1]. The statistic is defined below. The numerator is the number of concordant-pairs minus discordant-pairs between the two ranked lists, and the denominator is the total number of ways to choose two items from a rank-P list: $$\left(\begin{array}{c}P\\ 2\end{array}\right)$$.

$$\tau = \frac{{\#}_{concordant-pairs} - {\#}_{discordant-pairs}}{(P*(P-1)/2)}$$

Kendall’s tau, and other rank-based correlation metrics can be used to assess the quality/stability of either top-P word/token lists emerging from $\phi$; and can also be used to assess quality/stability of top-P document lists emerging from the matrix $\theta .$ An issue with Kendall’s tau in the context of comparing top-P lists is that the metric demands that all elements in one list be contained in the other list. In other words, the two top-P lists being compared must be conjoint, and not disjoint. Heuristics have been proposed to circumvent this challenge: for example, removing items/elements which occur in only one set from the scoring, or adding items occurring in only one set to the end of the other set. We have not seen these types of heuristics employed in the context of topic model evaluation; however, they are necessary, as bootstrap sampling does not ensure the same indices are present in even two pair-wise samples/models being compared. Hence, we consider concordance estimated over the intersection of indices present in two pairs of bootstrap samples. By the bootstrap 0.632 principle and the independence of the generated bootstrap samples, the concordance is estimated over approximately 0.632*0.632 = 0.399*100 percent of the original indices in the input DTM.

In this study, we use a variant of Kendall’s tau –weighted Kendall’s tau – and estimate concordance over all elements of $\theta$ and $\phi$, respectively. Using weighted Kendall’s tau, elements of the top-V or top-D lists are not given equal weight, rather items appearing higher in the ranked lists are given higher weighting under the weighted Kendall’s tau metric. Any positive weighting function can be employed – in this study we use the hyperbolic function: 1/(1 + r). The hyperbolic function assigns high weight to items appearing near the top of the ranked lists and attempts to ensure that arbitrary swaps/exchanges of low-ranking elements of the large lists do not unnecessarily deflate estimates of rank-based concordance.

### Stability analysis using fuzzy clustering quality indices

We note that NMF topic modelling resembles the framework of admixture/mixed-membership modelling [37]. The learned parameter matrices $(\theta$ and $\phi )$ contain non-negative real numbers; however, we can scale the row vectors of both $\theta$ and $\phi$ to represent probability/compositional vectors, dividing each by its respective row sum. Following transformation of the row-vectors from the $\theta$ matrix to the probability simplex, the NMF topic model closely resembles the grade of membership matrix used in fuzzy clustering models [38], and hence quality indices developed for validating fuzzy clustering solutions may be used to evaluate aspects of the quality of NMF topic model fits.

The normalized matrix of per-document topic-weights (θ^*^) describes the affinity of a given document for a specific latent topic vector. Each row of θ^*^ is a compositional/probability vector (living on K-1 dimensional simplex). Under this transformation, the matrix θ^*^ closely resembles the grade-of-membership matrix in fuzzy clustering models. Several validity indices from the fuzzy clustering community have been developed for investigating aspects of the fuzzy clustering solutions, including the (modified) partition coefficient (PC) and the (modified) partition entropy (PE) [39, 40]. Mathematical details of both validity indices are given below. Hard clustering solutions would represent θ^*^ as d = 1…D bit-vectors (i.e. K-1 elements would be 0, and a single element would equal 1). As fuzzy clustering solutions (and membership vectors) tend towards bit-vector type solutions, mixed-membership/admixture type models begin to look more like hard-clustering models (i.e. a data point is assigned to only a single latent topical vector; rather than a mixture over latent topics). This phenomenon is captured by the partition coefficient and partition entropy validity indices. For the partition coefficient, scores near 1 imply a hard-type clustering whereas, scores closer to zero imply a fuzzy clustering where documents spread topical prevalence weight across observed latent dimensions. Conversely, for the partition entropy, scores near zero imply a near hard clustering solution; whereas, positive scores indicate a more fuzzy clustering solution.

$$PC= \frac{1}{D}\sum_{d=1}^{D}\sum_{k=1}^{K}{{(\theta }_{\left\{d,k\right\}}^{*})}^{2}$$

$$PE= -\frac{1}{D}\sum_{d=1}^{D}\sum_{k=1}^{K}{\theta }_{\left\{d,k\right\}}^{*}\mathrm{log}({\theta }_{\left\{d,k\right\}}^{*})$$

The PC and PE validity indices discussed above focus only on evaluating the per-document topic-weight matrix (θ^*^). Alternative fuzzy clustering validity indices have been devised and reviewed [38]; many of the fuzzy clustering metrics attempt to evaluate aspects of cluster separation and compactness. The Xie-Beni index is mathematically described in [41, 42] and is one such fuzzy-cluster validity index which may be applied to assess quality of a transformed NMF topic model solution. Smaller values of the Xie-Beni index indicate a better fuzzy clustering model fit (i.e. topics are compact and well-separated; when scaled over the grade-of-membership matrix). As such, the Xie-Beni index is one metric which simultaneously evaluates aspects of both latent matrices ($\theta$ and $\phi$).

$$XB= \frac{\sum_{k=1}^{K}\sum_{d=1}^{D}{{(\theta }_{\left\{d,k\right\}}^{*})}^{2}{\Vert {X}_{d}-{\phi }_{k}\Vert }_{2}}{D* \underset{j\ne k}{\mathrm{min}}{\Vert {\phi }_{k}- {\phi }_{j}\Vert }_{2}} \approx \frac{(weighted) variance}{separation}$$

In terms of stability analysis, we can draw b = 1…B bootstrap replicate samples (with replacement) from the original DTM (X), and estimate the fuzzy cluster stability indices on NMF topic models fit to each bootstrap dataset and assess robustness/stability against data perturbations.

### Data augmentation based stability analysis

Many of the NMF topic model validity indices discussed above could be applied to stochastically augmented/perturbed versions of the original DTM (rather than bootstrap replicate data samples). In this scenario we envision the DTM being structured in (d,v,x) triplet/coordinate format – represented as (row-index, column-index, value) tuples. Using a data augmentation approach to stability analysis, one could maintain the row/column-indices in the coordinate format data structure, and stochastically augment the observed data value using random noise. One may draw an entirely new value from some parametric distribution; or they may jitter the observed data value (up/down) by some random value, while obeying constraints of the original data generating mechanism (e.g. DTM entries are non-negative count/integer random variables).

There exist certain advantages to the data augmentation approach to topic model stability analysis: 1) it is computationally much faster to sample/jitter new data values from the triplet/coordinate format sparse DTM than it is to draw legitimate cross-validation or bootstrap samples, and 2) data augmentation trivially ensures that the row/column indices from the original DTM are preserved and that documents and/or words/tokens appearing in the input DTM also appear in the augmented samples (this may not be the case with cross-validation or bootstrap sampling, and if desired must be verified via programmatic assertions). As a limitation, data augmentation may not be theoretically as principled a methodology for assessing model stability and robustness as compared with cross-validation and bootstrap resampling. Further, assumptions regarding the distribution generating augmented values are subjective. We do not experimentally investigate the suitability of data augmentation in NMF topic model validity, but we do consider it to be an interesting area for further research.

## Methods

### Study design, setting, data sources and inclusion/exclusion criteria

Our study employs a retrospective open cohort design. Encounter-level data are collected from patient primary care electronic medical records from Toronto, Canada. Data curation and cleaning is conducted by the University of Toronto Practice Based Research Network (UTOPIAN: https://www.dfcm.utoronto.ca/utopian). Additional details regarding UTOPIAN, including sampling, representativeness and data curation are given in Garies et al. [43]. The study start date is January 1, 2017 and the study end date is December 31, 2020. We include all patients who contribute at least one primary care clinical progress note during our study timeframe. We exclude patients who are missing basic demographic information (e.g. age or sex) or study identifiers (e.g. patient ID, physician ID, clinic ID).

### Computationally processing the clinical progress note corpus

#### Representing collections of text data using a document term matrix

A document term matrix is a D*V dimensional matrix. D represents the number of documents in the collection (here the number of unique clinical progress notes, written during patient-provider interactions). V represents the number of unique words/tokens in the empirical vocabulary of the document collection. A given element (X_{d,v}_) of the matrix, is a count random variable, denoting the number of times a particular word/token (v) was used in a particular document (d).

### Pre-processing text data

Raw clinical text data are a sequence of digital characters (letters, numbers, punctuation, other symbols, etc.). For each document in our collection, these raw text strings must be processed into individual words/tokens to facilitate creation of a DTM. Many approaches exist for processing clinical text data. We discuss several key elements of our text pre-processing pipeline, namely, tokenization, vocabulary normalization and dictionary/vocabulary creation.

Tokenization refers to the process of separating raw text strings (i.e. digital character sequences) into individual words/tokens [44, 45]. We employ a simple form of “whitespace tokenization”, which separates input character sequences into words/tokens based on the presence of whitespace boundaries (e.g. spaces, tabs, newlines, etc.).

Text normalization refers to the process of converting word/tokens into a single canonical form. We normalize tokens based on regular expressions, namely: case folding (lowercase conversion), and removal of punctuation/numbers.

Following tokenization and normalization we manually review the most frequently occurring words in the corpus and select to include 2210 tokens for inclusion in the corpus vocabulary; where the particular token-set selected consisted of focused/specific medical entities (e.g. disease names, disease symptoms, drug names, medical procedures, medical specialties, anatomical locations, etc.).

### Non-negative matrix factorization

We estimated NMF topic models using the module *sklearn.decomposition.NMF()* from *sklearn* version = 0.24.2 in 64-bit Python version 3.6. We varied model complexity (K = {5,10,25,50,75,100,150,200,250} topics) and investigated which models are selected as optimal using different metrics of model quality. We did not apply any regularization to latent parameter matrices. We randomly initialized parameter matrices. We estimated parameters using a gradient descent method on an L2/Frobenius-norm loss function, and we employed a loss function convergence tolerance of 1e-5 for terminating iterative update processes. 

### Experiments comparing the quality of NMF topic models on the Utopian clinical note corpus

A) Monte Carlo cross-validation using a reconstruction error metric: ${\Vert X- \sum_{k=1}^{K}{\theta }_{dk}{\phi }_{kv}\Vert }_{2}$
B) (Average) bootstrap stability using UCI/UMASS topic coherence metrics (over phi)
C) (Pair-wise average) bootstrap stability using a RBO metric (over phi)
D) (Pair-wise average) bootstrap stability using a RBO metric (over theta)
E) (Pair-wise average) bootstrap stability using Kendall’s tau metric (over phi)
F) (Pair-wise average) bootstrap stability using Kendall’s tau metric (over theta)
G) (Average) bootstrap stability using PC/PE fuzzy clustering coefficients (over theta)
H) (Average) bootstrap stability using XB fuzzy clustering metric (over theta and phi)

### Research ethics

This study received ethics approval from North York General Hospital Research Ethics Board (REB ID: NYGH #20–0014).

## Results

### Description of corpus

The study corpus/sample consists of 382,666 primary care progress notes from 44,828 patients, 54 physicians, and 12 clinics collected 01/01/2017 through 31/12/2020 from Toronto, Canada.

### NMF Per-topic word/token distribution

We summarize the corpus using the k = 1…K rows of the matrix $\phi .$ We report on the top-5 words loading most highly on each topical vector (Table 2). Next to each word/token in Table 2, we additionally display its probability of occurrence under topic k = 1…50. These topical vectors provide a low-dimensional thematic summarization of the clinical text dataset. We observe interesting topics corresponding to many major thematic areas of primary care, including: acute health conditions (e.g. COVID-19 and other respiratory conditions such as cough, flu, and colds), chronic physical health conditions (e.g. heart disease, cancer, arthritis and other musculoskeletal issues), mental health conditions (e.g. anxiety, depression and sleep issues), preventative health/screening (e.g. pap smears, flu shots, diet/exercise) and social/familial dynamics.

**Table 2:** Top-5 words/tokens loading most strongly on each of the learned k = 1…50 topical/thematic bases (row vectors of $\phi$), along with the per-topic word occurrence probabilities, from the fitted NMF model (with K = 50 latent bases)

	Word 1	Word 2	Word 3	Word 4	Word 5
Topic 1	tylenol (34.7)	advil (9.1)	tab (2.6)	headache (2.5)	tabs (2.3)
Topic 2	mg (44.8)	tab (2.1)	tabs (1.7)	capsules (1.4)	po (1.3)
Topic 3	fever (33.9)	diarrhea (2.5)	vomiting (2.4)	tylenoladvil (2.3)	viral (2.1)
Topic 4	neck (21.1)	head (4.2)	arm (2.8)	headache (2.3)	headaches (2.0)
Topic 5	bw (31.2)	iron (3.2)	tsh (2.3)	ferritin (1.9)	thyroid (1.7)
Topic 6	work (46.7)	social (4.5)	stress (3.4)	working (3.2)	treatment (2.7)
Topic 7	bp (57.8)	systolic (3.7)	diastolic (3.3)	htn (2.7)	norvasc (1.5)
Topic 8	sleep (36.9)	bed (4.6)	sleeping (3.2)	apnea (2.5)	insomnia (2.2)
Topic 9	anxiety (30.4)	anxious (4.3)	panic (3.1)	social (2.5)	counselling (2.4)
Topic 10	flu (36.7)	shot (31.9)	anaphylactic (3.0)	influenza (2.8)	ibuprofen (2.0)
Topic 11	weight (32.1)	kg (9.0)	bmi (4.7)	height (3.7)	lbs (2.8)
Topic 12	pain (51.7)	palpation (1.7)	flexion (1.2)	physio (1.0)	arm (1.0)
Topic 13	ear (31.0)	hearing (5.7)	ears (5.3)	wax (4.9)	cerumen (4.8)
Topic 14	eating (4.9)	diet (4.3)	food (3.6)	wt (3.2)	snack (2.7)
Topic 15	throat (23.3)	sore (12.8)	strep (4.0)	viral (2.8)	nodes (2.6)
Topic 16	rx (43.4)	shingrix (1.4)	ativan (1.3)	ra (1.3)	abx (1.2)
Topic 17	meds (43.1)	bmd (1.3)	vit (1.2)	chronic (1.1)	bone (1.1)
Topic 18	pap (12.4)	bleeding (3.8)	vaginal (2.6)	discharge (2.4)	pelvic (2.3)
Topic 19	vaccine (20.5)	influenza (8.2)	flu (7.8)	allergy (5.6)	fever (5.2)
Topic 20	dose (31.3)	medication (9.8)	immunization (5.0)	injection (3.8)	shingrix (2.7)
Topic 21	breast (26.7)	cancer (3.2)	nipple (3.0)	mammogram (2.4)	lump (2.2)
Topic 22	medications (14.6)	allergy (6.4)	drug (5.0)	capsules (5.0)	capsule (4.7)
Topic 23	cough (25.7)	sob (3.1)	ventolin (2.9)	asthma (2.6)	coughing (2.5)
Topic 24	bilat (26.4)	masses (2.1)	neuro (1.9)	limbs (1.8)	head (1.7)
Topic 25	heart (19.7)	bpm (17.2)	systolic (16.2)	diastolic (16.0)	bp (2.4)
Topic 26	urine (13.6)	uti (6.8)	urinary (4.3)	dysuria (3.8)	hematuria (3.4)
Topic 27	eye (27.5)	vision (5.6)	drops (4.9)	eyes (4.5)	discharge (3.9)
Topic 28	symptoms (41.6)	nausea (1.6)	urinary (1.6)	headache (1.4)	gi (1.2)
Topic 29	foot (11.9)	swelling (7.3)	ankle (4.2)	toe (3.7)	feet (2.3)
Topic 30	sx (41.1)	neuro (3.0)	gi (2.5)	urinary (2.0)	melena (1.5)
Topic 31	mother (29.6)	father (5.5)	parents (2.1)	sister (2.0)	mothers (1.6)
Topic 32	mood (22.3)	cipralex (4.3)	depression (3.1)	counselling (3.0)	speech (2.7)
Topic 33	exercise (6.1)	diet (5.0)	ldl (3.4)	screening (2.0)	cancer (2.0)
Topic 34	tablets (27.3)	tablet (26.4)	medications (7.0)	oral (4.6)	mg (3.9)
Topic 35	rn (24.1)	immunization (3.4)	injection (2.8)	baby (2.4)	arm (1.8)
Topic 36	er (23.9)	felt (5.3)	head (3.5)	ct (3.4)	sob (2.4)
Topic 37	covid (22.7)	health (13.6)	physical (13.3)	emergency (10.5)	pandemic (4.1)
Topic 38	back (48.7)	spine (2.1)	lumbar (1.7)	flexion (1.7)	physio (1.5)
Topic 39	mom (36.2)	dad (2.9)	parents (2.0)	baby (1.8)	feeding (1.4)
Topic 40	chest (27.5)	sob (4.2)	cvs (3.0)	edema (1.9)	palpitations (1.8)
Topic 41	knee (28.7)	swelling (4.7)	oa (3.2)	joint (2.6)	medial (2.5)
Topic 42	blood (30.9)	pressure (13.6)	medication (3.0)	pulse (2.6)	pounds (2.2)
Topic 43	family (8.2)	social (5.8)	counselling (4.2)	husband (4.0)	daughter (3.8)
Topic 44	feeling (39.6)	felt (4.6)	tired (2.8)	anxious (2.6)	treatment (1.6)
Topic 45	feels (50.4)	felt (2.5)	tired (1.3)	stress (1.3)	anxious (1.2)
Topic 46	hip (23.4)	xray (4.8)	oa (3.4)	physio (2.6)	flexion (2.0)
Topic 47	nasal (19.1)	sinus (6.0)	congestion (5.8)	nose (3.8)	nasonex (3.4)
Topic 48	skin (13.1)	rash (8.5)	cream (4.3)	derm (3.3)	lesions (2.9)
Topic 49	referral (32.4)	derm (3.2)	ent (1.8)	gi (1.6)	mri (1.5)
Topic 50	abdo (13.5)	diarrhea (3.6)	stool (3.3)	bm (2.9)	masses (2.6)

Human judgement validation was used to determine the complexity of the model below (i.e. K = 50 latent topical bases). On inspection, we found that a model with approximately K = 50 latent topical dimensions resulted in a parsimonious summary of the primary care clinical text corpora. Models with far fewer topics often resulted in an incomplete summarization of primary care topics; and/or resulted in distinct primary care concepts being grouped under a single topical construct. Conversely, models with a far greater number of topics were often more time consuming to interpret, and resulted in semantically similar topics being redundantly described. In the results sub-sections which follow, we compare topic model complexity identified via human judgement evaluation, with those identified using quantitative topic model quality indices.

### NMF Per-document topic-distribution

We inspected the top-5 documents loading most highly on each of the k = 1…K columns of the latent matrix $\theta .$ Excerpts of the most-relevant documents under each topical query provide complementary evidence that the learned latent basis effectively summarizes the corpus, and further can be used as a tool to facilitate document retrieval and clustering. We observed that documents loading most highly on a topical vector of $\theta$ are semantically related to the corresponding word/tokens used to describe the topic (Table 2). Top-5 most probable documents under a particular topic are not displayed because clinical text excerpts may contain sensitive information and/or other protected health information.

### NMF Topic model validity indices

In the subsections below we apply several topic model quality indices to the primary care clinical note corpus. We demonstrate how different topic model quality indices highlight different aspects of model goodness of fit, stability, and robustness.

### Monte Carlo cross-validation using a (predictive) reconstruction error metric

**Fig. 1:**
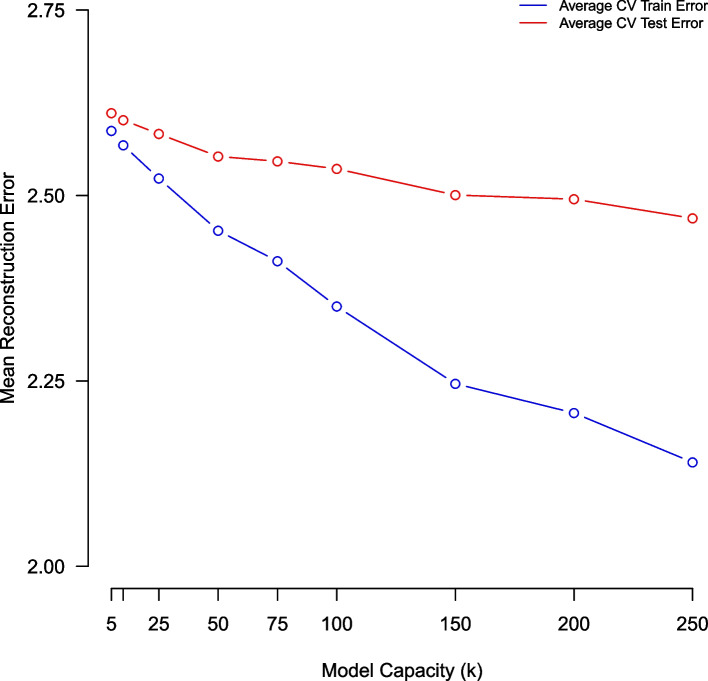
Average training/testing reconstruction error of NMF models of complexity K = (5,10,25,50,100,150,200,250) estimated using five-fold Monte Carlo cross-validation

In this subsection we use a Monte Carlo cross-validation methodology for comparatively evaluating topic model quality, across NMF models of complexity K = (5, 25, 50, 75, 100, 150, 200, 250). For each model complexity parameter (k) we assess mean reconstruction error on training and held-out test samples, using a five-fold Monte Carlo cross-validation scheme. The results suggest that larger (more complex) NMF models provide a more optimal fit to the primary care corpus (Fig. 1).

### (Average) bootstrap stability using UCI/UMASS topic coherence metrics (over phi)

**Fig. 2:**
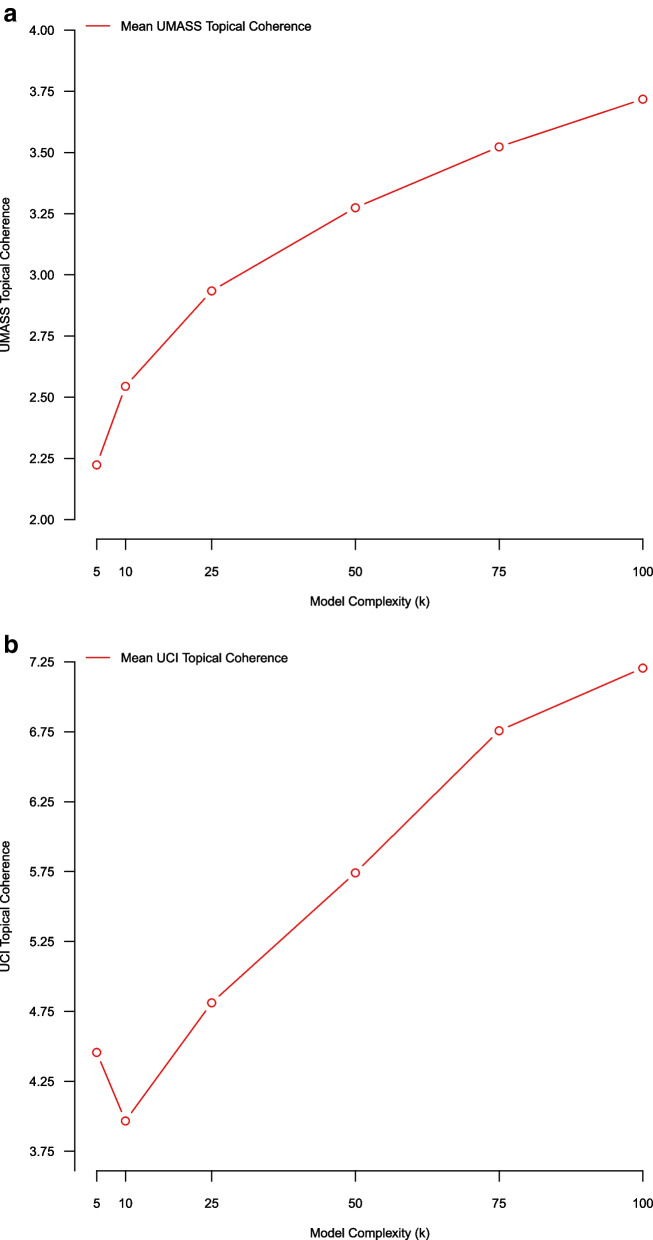
Average topic coherence of NMF models of complexity K = (5,10,25,50,75,100) estimated using five-fold stability analysis. The left-hand panel plot uses the UCI topic coherence score, and the right-hand panel plot uses the UMASS topic coherence score

In this subsection we use an average bootstrap stability analysis methodology employing a topic coherence metric for assessing model quality over NMF complexity parameters K = (5, 10, 25, 50, 75, 100). For a given NMF model of complexity K, we average the k = 1…K topical coherences vectors (${\phi }_{k})$, resulting in a single measure of model coherence. We further compare model-based topical coherence scores across the five separate bootstrap replicate samples in the stability analysis, and observe higher scores at larger values of model complexity (implying more coherent topics) (Fig. 2).

### (Pair-wise average) bootstrap stability using a RBO metric (over phi)

**Fig. 3:**
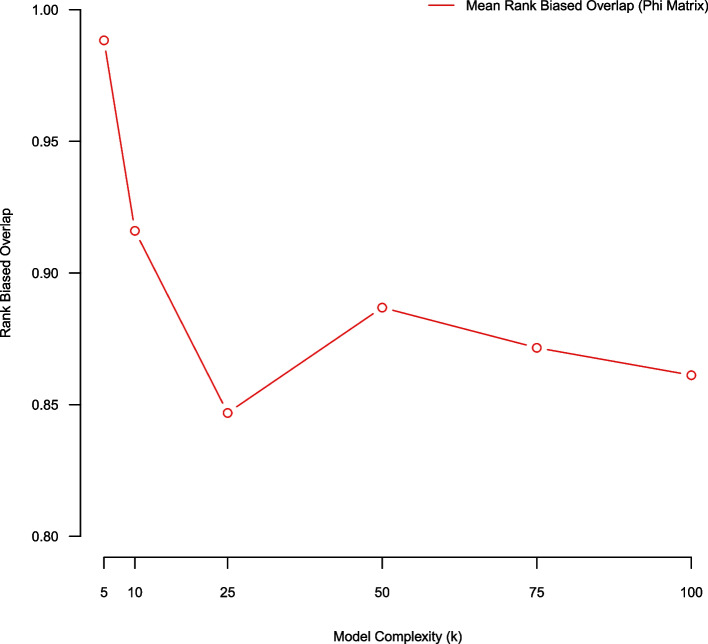
Average rank-biased overlap of $\phi$ over NMF models of complexity K = (5,10,25,50,75,100) estimated using five-fold stability analysis

In this subsection we use an average bootstrap stability analysis methodology employing a rank biased overlap metric for assessing model quality over NMF complexity parameters K = (5, 10, 25, 50, 75, 100). Using a five-fold stability analysis there exist $$\left(\begin{array}{c}5 \\ 2\end{array}\right)$$ pair-wise model comparisons (over aligned NMF topical matrices $\phi ).$ We average the pair-wise RBO scores from the five-fold stability analysis process and comparatively evaluate model quality over complexity K = (5, 10, 25, 50, 75, 100). Using RBO we favor smaller models (K = {5,10}) (Fig. 3).

### (Pair-wise average) bootstrap stability using a RBO metric (over theta)

**Fig. 4:**
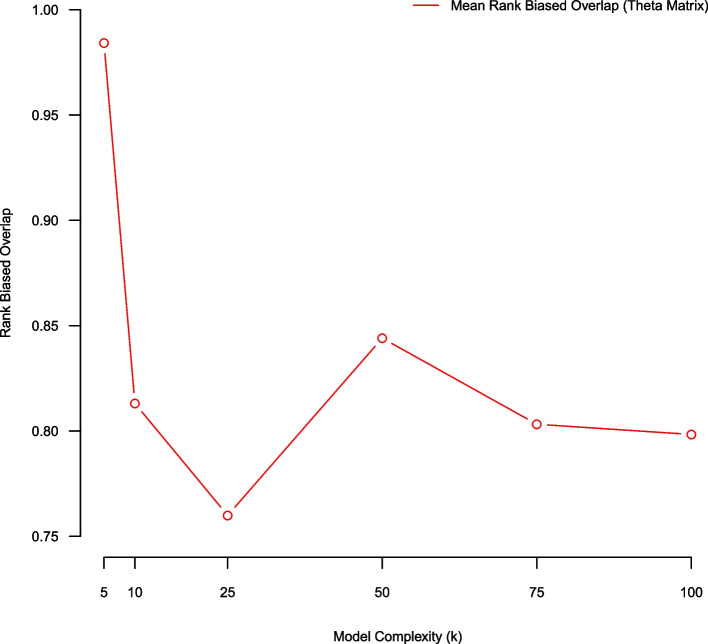
Average rank-biased overlap of $\theta$ over NMF models of complexity K = (5,10,25,50,75,100) estimated using five-fold stability analysis

In this subsection we use an average bootstrap stability analysis methodology employing a rank biased overlap metric for assessing model quality over NMF complexity parameters K = (5, 10, 25, 50, 75, 100). Using a five-fold stability analysis there exist $$\left(\begin{array}{c}5 \\ 2\end{array}\right)$$ pair-wise model comparisons (over aligned NMF per-document topic-prevalence matrices $\theta ).$ We average the pair-wise RBO scores from the five-fold stability analysis process and comparatively evaluate model quality over complexity K = (5, 10, 25, 50, 75, 100). Using RBO we favor smaller models (K = {5,10}) (Fig. 4).

### (Pair-wise average) bootstrap stability using weighted Kendall’s tau metric (over phi)

**Fig. 5:**
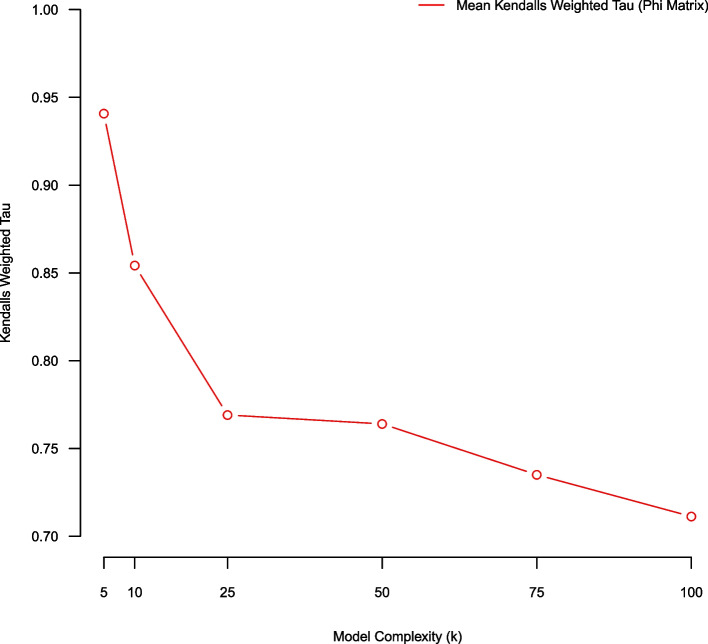
Average Kendall’s weighted tau of $\phi$ over NMF models of complexity K = (5,10,25,50,75,100) estimated using five-fold stability analysis

In this subsection we use an average bootstrap stability analysis methodology employing a weighted Kendall’s tau metric for assessing model quality over NMF complexity parameters K = (5, 10, 25, 50, 75, 100). Using a five-fold stability analysis there exist $$\left(\begin{array}{c}5 \\ 2\end{array}\right)$$ pair-wise model comparisons (over aligned NMF topical matrices $\phi ).$ We average the pair-wise Kendall weighted tau statistics from the five-fold stability analysis process and comparatively evaluate model quality over complexity K = (5, 10, 25, 50, 75, 100). Using Kendall’s weighted tau we favor smaller models (K = {5,10}) (Fig. 5).

### (Pair-wise average) bootstrap stability using weighted Kendall’s tau metric (over theta)

**Fig. 6:**
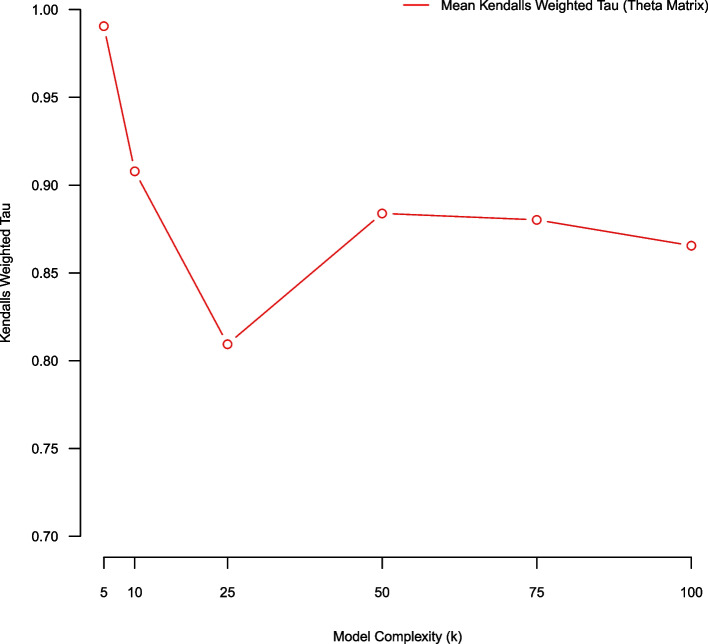
Average Kendall’s weighted tau of $\theta$ over NMF models of complexity K = (5,10,25,50,75,100) estimated using five-fold stability analysis

We used an average bootstrap stability analysis methodology employing a weighted Kendall’s tau metric for assessing model quality over NMF complexity parameters K = (5, 10, 25, 50, 75, 100). Using a five-fold stability analysis there exist $$\left(\begin{array}{c}5 \\ 2\end{array}\right)$$ pair-wise model comparisons (over aligned NMF topical matrices $\theta ).$ We average the pair-wise Kendall weighted tau statistics from the five-fold stability analysis process and comparatively evaluate model quality over complexity K = (5, 10, 25, 50, 75, 100). Using Kendall’s weighted tau we favor smaller models (K = {5,10}) (Fig. 6).

### (Average) bootstrap stability using PC/PE fuzzy clustering coefficients (over theta)

**Fig. 7:**
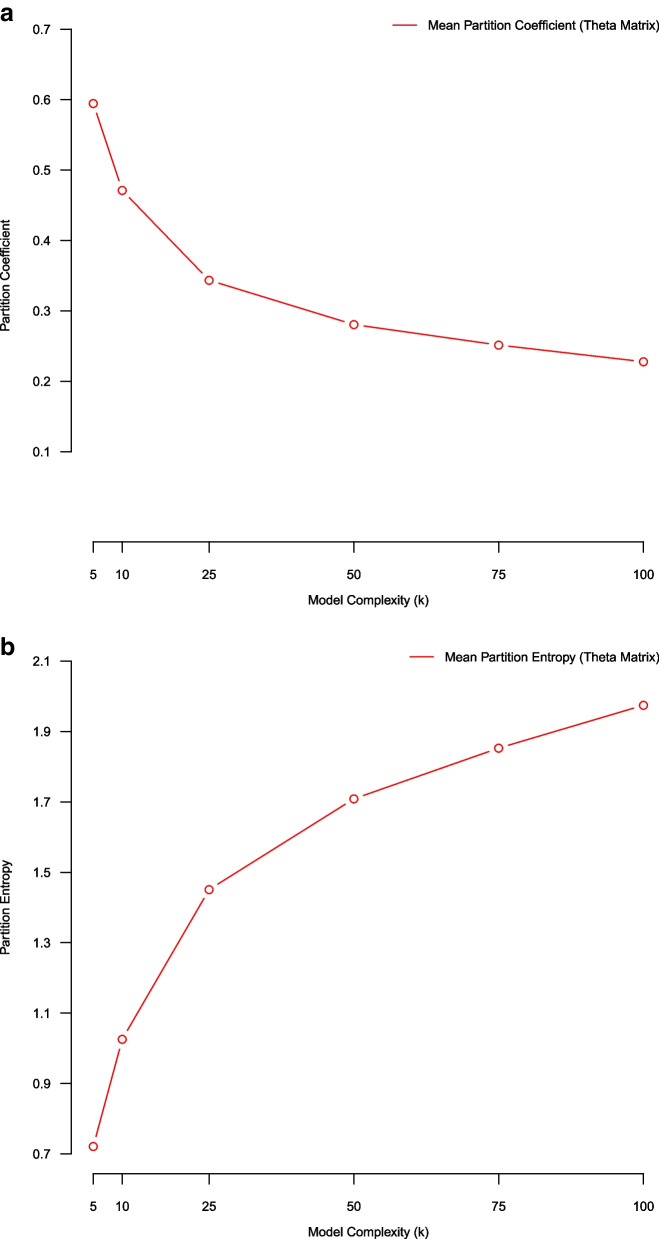
Average partition coefficient and partition entropy scores of NMF models of complexity K = (5,10,25,50,75,100) estimated using five-fold stability analysis. The left-hand panel plot uses the partition coefficient score, and the right-hand panel plot uses partition entropy score. Both the partition coefficient and the partition entropy suggest that smaller models result in more “crisp” clustering solutions; whereas, larger models result in more “fuzzy/admixed” clustering solutions

In this subsection we use an average bootstrap stability analysis methodology employing partition coefficient and partition entropy metrics for assessing model quality over NMF complexity parameters K = (5, 10, 25, 50, 75, 100). For a given NMF model of complexity K we average/compare partition coefficient/entropy scores across the five separate bootstrap replicate samples in the stability analysis (Fig. 7).

### (Average) bootstrap stability using Xie-Beni fuzzy clustering metric (over theta and phi)

**Fig. 8:**
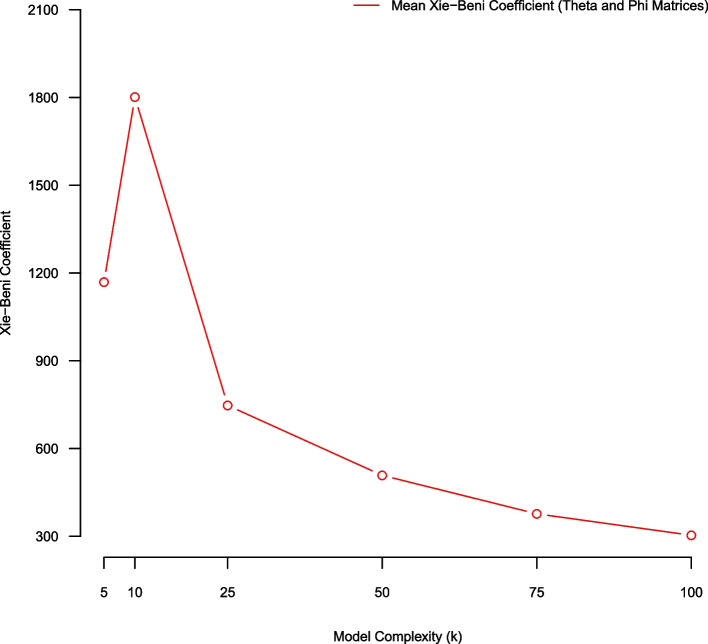
Average Xie-Beni scores of NMF models of complexity K = (5,10,25,50,75,100) estimated using five-fold stability analysis

In this subsection we use an average bootstrap stability analysis methodology employing Xie-Beni fuzzy clustering metrics for assessing model quality over NMF complexity parameters K = (5, 10, 25, 50, 75, 100). For a given NMF model of complexity K we average/compare Xie-Beni scores across the five separate bootstrap replicate samples in the stability analysis. The Xie-Beni statistic appears to favor larger topic model fits on our observed corpus (Fig. 8).

## Discussion

The major finding of this study (and one which has been observed elsewhere) is that different topical model quality indices do not necessarily agree on a single topic model as being “optimal” when applied to a given empirical dataset. Cross-validated reconstruction error, (averaged) topic coherence and (averaged) Xie-Beni score (a ratio of compactness vs separation) were observed to favour large models; whereas, set based agreement measures (rank biased overlap) and rank correlation measures (weighted Kendall’s tau) pair-wise averaged over aligned bootstrapped datasets seem to favour small models. Few/none of the metrics were observed to guide selection towards “mid-sized” models as being optimal (which were subjectively preferred based on human/analyst judgement). Large models (K ≥ 100 topics) were observed to produce smaller residual error (we do not observe an increase in validation/test error even at K = 250 topics; noting that fitting larger models is computationally prohibitive on our dataset). Similarly, large models were observed to generate focused, interpretable, and coherent topical vectors. And finally, the Xie-Beni fuzzy clustering coefficient is suggestive that geometrically compact topics/clusters that are well-separated form at increasing model complexity. On the contrary, stability metrics based on rank correlation (e.g. weighted Kendall’s tau) and set based agreement (rank biased overlap) seem to favour models of much lower complexity (K = 5 topics). The evidence suggests that different topic model quality indices lead to different inferences regarding an optimal NMF topic model. The investigated quality indices provide different/complementary insights regarding model goodness of fit, and it likely makes sense to utilize numerous indices when evaluating fitted models to empirical datasets.

Using a human-centric approach to NMF topic model selection, where data scientists and subject matter experts attempt to select an optimal topic model fitted to the primary care clinical note corpus (after “eyeballing” fits at k = {5,10,25,50,75,100}), we prefer mid-sized models (K = 50 topic model). The mid-sized models (qualitatively) satisfy many desirable properties of a topic model: 1) the latent topical basis provide a meaningful characterization of the document collection, facilitating an improved understanding of the large/complex primary care progress notes corpus, 2) the document topic weights seem appropriate, providing an efficient low-dimensional basis for retrieval, clustering and browsing of documents, and 3) the model explained variation is reasonable (although could be improved with a more complex model). In terms of human judgement, we find large NMF topic models (subjectively) to be overly complex. It takes a great deal of time/effort to meaningfully “eyeball” K ≥ 100 topical bases (and high-loading documents). Further, when an NMF model of excessive complexity is fit to the primary care progress note corpus we observe an over-clustering effect where many of the learned focused/specific topics appear redundant (and by Ockham’s razor, this may suggest a more parsimonious model is attainable and perhaps preferrable). On the contrary, we find that the NMF topic models of low complexity (K = {5,10}) which are preferred by the rank correlation metrics and the set based agreement metrics are not expressive enough to adequately summarize a complex clinical text corpus (subjectively, the space of primary care medicine is about more than 5–10 topicals/themes).

### Limitations and future work

We focused on several popular quality indices applicable to the evaluation of NMF topic models; however, our review is necessarily incomplete (as metrics are disparately studied over a vast number of scientific disciplines) and our evaluation focuses on a single large/complex biomedical text corpus. Future work should attempt to further synthesize/consolidate an increasing number of topic model quality indices, and further evaluate these metrics over many real and simulated datasets. Formal systematic literature reviews and/or scoping reviews may be valuable, as they could more exhaustively identify the space of available topic model quality indices. That said, our review has focused on some of the more popular metrics from a variety of disparate fields, such as: computer science and topic modelling, matrix factorization, fuzzy clustering, and set theory.

This study focused on evaluating/selecting an optimal model complexity parameter (K) over fitted NMF topic models. Metrics presented above could also be used to investigate other NMF model hyper-parameters, for example: model loss function, model functional form, regularization, initialization techniques, and model termination criteria are all relevant hyper-parameters whose optimal configuration can be assessed with quality indices discussed in this study. Incorporation of the aforementioned topic model quality indices, in a formal hyper-parameter optimization framework, may help to guide the analyst towards an optimal hyper-parameter configuration for a topic model fitted to a particular empirical dataset [46].

This study has focused on combining appropriate topic model quality metrics with computational resampling methods (e.g. cross-validation or bootstrapping) for assessing NMF topic model goodness of fit. The evaluation pipeline is computationally expensive: Monte Carlo cross-validation requires specific checks on the validity of returned DTMs and stability analysis may require application of expensive matrix alignment methods. Data augmentation was not thoroughly explored in this study; however, it may represent an interesting and computationally affordable approach to topic model evaluation.

This study focused on quality indices for evaluating aspects of NMF topic models. We did not compare NMF topic model fits against alternative topic modelling frameworks, for example: Bayesian probabilistic graphical models (e.g. latent Dirichlet allocation), neural topic models (e.g. BERTopic) or tensor factorization models (e.g. the canonical polyadic decomposition, or the Tucker decomposition). Each of the aforementioned methodologies estimate a latent representation, characterizing the extent to which 1) words load on topical vectors, and 2) document load on topical vectors. As such, many of the topic model quality indices investigated in this study could be used to evaluate topic models generated using alternative approaches to statistical estimation.

In this study we adopted a hybrid approach to text processing and vocabulary construction. First, we performed an initial computational tokenization pass over the corpus; next, we reviewed the returned list of tokens and a human determined which ones to include in the final vocabulary (focusing particularly on lexical entities relevant to primary healthcare). The number of unique (and justifiable) approaches to text processing are essential uncountably large. This study did not investigate alternative text processing pipelines, and their impact on topic model quality. For example, we did not consider using stemmers/lemmatizers; nor did we attempt to group semantically similar lexical variants post-tokenization. Further research should continue to investigate the impact of text processing pipelines on vocabulary specification in vector space models, and in particular topic models.

## Conclusions

In this study we reviewed and comparatively evaluated several topic model quality indices. Oftentimes an eyeballing approach is used in topic model selection/evaluation, whereby subject matter experts and data scientists iteratively review learned topic models and subjectively determine an appropriate fitting model for the corpus at hand – the approach is often criticized as lacking empirical rigor, and advocates often suggest employing one of potentially many available topic model quality indices for guiding model selection. This study illustrates some challenges associated with the latter line of thought, namely, a large host of defensible topic model quality indices exist, and the choice of an optimal model appears metric dependent (i.e. different quality metrics guide the analyst toward fundamentally different NMF topic models). This finding does not invalidate quantitative topic model quality indices, rather it suggests that different metrics highlight different aspects of model goodness of fit. Further, human in the loop approaches to topic model selection/evaluation are likely still required where different models (under different hyper-parameter configurations) are fitted to empirical datasets, and evaluated using a combination of human judgment in addition to different quality indices. Both quantitative topic model quality indices, and human judgement evaluation, are crucially important when interpreting unsupervised machine learning models.

## Supplementary Information

**Additional file 1. **Appendix A and B.

## Data Availability

Data from this study are held by the University of Toronto Practice-Based Research Network. Ethics approval for this study does not allow access to patient level data outside of the trusted research environment in which it is held. Researchers can apply for access to the data, subject to approval by appropriate research ethics boards, and approval of the UTOPIAN scientific advisory committee.
