# Supplementary material for: Quality indices for topic model selection and evaluation: a literature review and case study
Source: BMC Med Inform Decis Mak. 2023 Jul 22;23:132. doi: 10.1186/s12911-023-02216-1 (PMC10362613; doi:10.1186/s12911-023-02216-1)
Supplement: Supplementary file 1 — Additional file 1. Appendix A and B. [file 12911_2023_2216_MOESM1_ESM.docx]

**Appendix A**

***Linear Sum Assignment Algorithms for Matrix Alignment (via the Hungarian Algorithm):***

To compare ranked lists arising from 1) the matrix of per-document topical weights ($\theta$), or 2) the matrix of per-topic word/token probabilities ($\phi$) from a fitted NMF model, we must first align the respective matrices under consideration. Pairs of topical matrices are compared under a bootstrap stability analysis; however, topical vectors arising from independent NMF fits are exchangeable up to arbitrary permutations. To ensure we are applying topic model quality indicators (e.g. set based agreement measures or weighted concordance statistics) over semantically similar topical vectors, we must first align the returned matrices arising from independent bootstrap stability analysis fits, which can be accomplished using a linear sum assignment algorithm (often called the Hungarian algorithm) [34,35].

Consider two rectangular matrices $A \in R^{K,V}$ and $B \in R^{K,V}.$ The dimensions of A and B respectively are chosen to be analogous to the right-hand topical/thematic summarization vectors arising from a fitted NMF topic model. Further consider a square permutation matrix $\pi\in R^{K,K}$(n.b. a permutation matrix is a square matrix with a single one in each column, a single one in each row, and zeros elsewhere). The goal is to solve the following optimization problem, aligning the rows of B such that the permutation-aligned matrix is as close to the original matrix A as possible (where closeness is measured in terms of Frobenius norm error).

$$\underset{\pi\epsilon\Pi}{\mathrm{argmin}} \left\| A- \pi B \right\|_{2}$$

Using properties of the Frobenius norm, we can re-write the optimization problem as:

$$\underset{\pi\epsilon\Pi}{\mathrm{argmin}} \left\| A- \pi B \right\|_{2}= \underset{\pi\epsilon\Pi}{\mathrm{argmin}} {(\left\| A \right\|}_{2}- 2*\left\langle A,\pi B \right\rangle+\left\| \pi B \right\|_{2})= \underset{\pi\epsilon\Pi}{\mathrm{argmin}}(-2*{\mathrm{trace}(AB^{T}\pi}^{T}))$$

The above minimization problem can be expressed as a maximization problem (by dropping negation). Further, we can drop arbitrary constants to simplify the optimization problem. And we can define a new cost matrix: $C \equiv AB^{T}.$

$$\underset{\pi\epsilon\Pi}{\mathrm{argmax}}\left( \mathrm{trace}\left( {AB^{T}\pi}^{T} \right) \right)= \underset{\pi\epsilon\Pi}{\mathrm{argmax}}\left( \mathrm{trace}\left( {C\pi}^{T} \right) \right)$$

We can determine the optimal permutation $\pi$ by relaxing the above maximization problem; expressing as a matching/linear-assignment problem (involving a cost matrix C and a permutation/assignment matrix $\pi$). This problem can be solved using the Hungarian algorithm [34,35]. We use the Python Scipy implementation: scipy.optimize.linear_sum_assignment().

**Appendix B**

**Text Pre-Processing Methodology for Vocabulary Specification and Document Term Matrix Construction**

Vector space models – such as the document term matrix (DTM) used in empirical topic modelling – rely crucially on the judicious specification of the study vocabulary. The study vocabulary refers to a set of discrete words/tokens, used to define the column space of the DTM.

Originally, the study corpus is structured as a list of d=1…D variable length character sequences (i.e. string variables); one for each document in the study corpus. Vocabulary specification in vector space models involves defining a mapping from a collection of d=1…D string variables to a discrete set of v=1…V words/tokens. There exists an essentially infinite space of algorithms for constructing such a mapping from strings to words/tokens. Popular strategies for vocabulary specification include: 1) computational methodologies relying on tokenizers, normalization routines, and other algorithms from natural language computing, 2) human-centric approaches, relying on expert derived dictionaries or ontologies, and 3) hybrid techniques, which utilize both computational tools and human knowledge.

The two primary objectives that informed our approach to text pre-processing – and vocabulary specification were 1) the method needed to be reasonably computationally efficient as well as reliable/trustworthy, and 2) the amount of work associated with human post-processing should be relatively minimal. These two general principles guided specification of our tokenization/normalization pipeline – and ultimately our approach to vocabulary specification. To be clear, our study adopted a hybrid approach to vocabulary specification – and in many clinical topic modelling settings, we would anecdotally argue that such an approach is relatively efficient, straightforward, and effective.

The initial approach to tokenization, normalization and vocabulary specification was purely computational. In particular, we began by computationally tokenizing the input digital character sequences on whitespace boundaries (i.e. space, tab, or newline characters). We normalized the resulting token set using lowercase conversion, removed all non-alphabetic characters from the resulting tokens, and removed tokens corresponding to common stop words. Next, we manually reviewed the top-10k most frequently occurring tokens resulting from the initial computational tokenization/normalization pass. Our goal in the manual human-review was to include semantically precise words/tokens of direct relevance to primary care medicine, while excluding all other words/tokens. The first set of exclusions removed frequently occurring words/tokens including: 1) common English stop words, 2) “medical stop words”, associated with descriptions of the patient-physician clinical interactions, that otherwise contained limited meaning in the context of primary care medicine (e.g. “doctor”, “patient”, “presented”, “complained”, “returned”, “scheduled”, “visit”, “encounter”, “clinic”, etc.), 3) dates and times, 4) mentions of the patient, their family and others involved in the circle of care, and 5) mentions of clinical setting (e.g. clinic, hospital, department, etc.). The second set of exclusions focused on low frequency words/tokens (i.e. all words/tokens outside of the top-10k most frequently occurring words/tokens). Certainly, some of the low frequency words were medical in nature, and their removal was a “false removal”. However, many of the words were likely misspellings, idiosyncratic acronyms, and tokenization errors. Ultimately, the final vocabulary used in our study included V=2210 words/tokens, all of which had precise semantic meaning in terms of primary care medicine (e.g. disease conditions and symptoms, pharmaceutical agents, medical procedures, clinical specialties, physiological/anatomical terms, etc.).

As mentioned above, there exist many defensible methodologies for vocabulary specification in vector space modelling, and applied topic modelling. Many subjective decisions were made in defining the hybrid computational/human-in-the-loop pipeline used to map digital character sequences to discrete words/tokens in our study. Below we comment on several subjective decisions we made.

First, we used a white-space tokenizer and relied on subjective normalization processes to map digital character sequences into discrete linguistic units (i.e. tokens). Several alternative choices of tokenization and normalization algorithms could have been employed – few of which have been studied on clinical/biomedical text data. An often-cited critique was that no stemming/lemmatization algorithms were employed. This decision was made because of observed variations in root forms identified across different stemming/lemmatization algorithms. We argue that more research is needed to better understand the relationship between tokenization, normalization, stemming and lemmatization algorithms in the context of processing clinical text data for use in topic modelling studies.

Second, our use of human knowledge to define words/tokens relevant to primary care medicine was also highly subjective. It should be noted – what is deemed as an important word/token must be defined in relation to the underlying study-specific research question. In this study, our goal was to estimate latent topical vectors defining archetypical primary care patients, processes, and procedures. We reviewed the top-10k most frequently occurring words/tokens (i.e. words with an occurrence frequency of approximately >50 in our empirical study corpus). This decision was driven by human resource constraints. With more time/money we may have been able to review additional words/token for inclusion/exclusion from the study. We focused on including only words/tokens with precise semantic meaning related to primary care medicine (e.g. disease conditions and symptoms, pharmaceutical agents, medical procedures, clinical specialties, physiological/anatomical terms, etc.). The judicious inclusion of semantically meaningful words/tokens relevant to our study research question was one of the main reasons that the learned topic model was able to successfully summarize our study corpus and facilitate browsing and retrieval from the large unstructured document collection. A related strategy (ultimately not employed in our study) could have involved the use a priori developed dictionaries/ontologies. Again, few dictionaries/ontologies of relevant words/tokens exist in the context of primary care medicine; hence we adopted the hybrid approach specified above. Further, we did not conduct any human-in-the -loop word/token normalization. For example, we could have post-hoc grouped multiple distinct words/tokens into a single semantic grouping, rather than analyze them as individual discrete words/tokens (e.g. diab, diabetes, dm2, dm1, diabetologist, etc. could all be grouped into a diabetes topic). In terms of methodology, we could have employed multiple human reviewers, assessed agreement between reviewers, and possibly incorporated a separate adjudication stage.

Little research has been conducted evaluating the impacts that text pre-processing pipelines have on downstream vector space models, and in particular empirical topic modelling. What limited research exists, often has occurred in the context of regular English texts and has not been evaluated in the context of more complex clinical/biomedical text data. Future research should continue to investigate the interplay between text pre-processing pipelines and inferences derived from studies employing topic models fit to clinical text corpora.
